# The Ubiquitination System within Bacterial Host–Pathogen Interactions

**DOI:** 10.3390/microorganisms9030638

**Published:** 2021-03-19

**Authors:** Vera Vozandychova, Pavla Stojkova, Kamil Hercik, Pavel Rehulka, Jiri Stulik

**Affiliations:** 1 Department of Molecular Pathology and Biology, Faculty of Military Health Sciences, University of Defence, Trebesska 1575, 50001 Hradec Kralove, Czech Republic; vera.vozandychova@unob.cz (V.V.); pavla.stojkova@gmail.com (P.S.); kamil.hercik@unob.cz (K.H.); pavel.rehulka@unob.cz (P.R.); 2 Institute of Organic Chemistry and Biochemistry of the Czech Academy of Sciences, Flemingovo namesti 542/2, 16000 Prague, Czech Republic

**Keywords:** ubiquitination, deubiquitinating enzymes (DUBs), effector protein, host–pathogen interaction

## Abstract

Ubiquitination of proteins, like phosphorylation and acetylation, is an important regulatory aspect influencing numerous and various cell processes, such as immune response signaling and autophagy. The study of ubiquitination has become essential to learning about host–pathogen interactions, and a better understanding of the detailed mechanisms through which pathogens affect ubiquitination processes in host cell will contribute to vaccine development and effective treatment of diseases. Pathogenic bacteria (e.g., *Salmonella enterica*, *Legionella pneumophila* and *Shigella flexneri*) encode many effector proteins, such as deubiquitinating enzymes (DUBs), targeting the host ubiquitin machinery and thus disrupting pertinent ubiquitin-dependent anti-bacterial response. We focus here upon the host ubiquitination system as an integral unit, its interconnection with the regulation of inflammation and autophagy, and primarily while examining pathogens manipulating the host ubiquitination system. Many bacterial effector proteins have already been described as being translocated into the host cell, where they directly regulate host defense processes. Due to their importance in pathogenic bacteria progression within the host, they are regarded as virulence factors essential for bacterial evasion. However, in some cases (e.g., *Francisella tularensis*) the host ubiquitination system is influenced by bacterial infection, although the responsible bacterial effectors are still unknown.

## 1. Introduction

Competition between host defense mechanisms and pathogens’ effective tools has been observed since ancient times. The immune system of the host organism is regulated by complex metabolic and signaling pathways, including, but not limited to, a network of post-translational modifications. Should any of these important pathways be impaired, such as by efficient mechanisms of pathogens, the immune balance is disrupted and pathogens may become more successful. During their development, pathogenic organisms have acquired several mechanisms by which they can specifically influence the immune response of the host and thus escape their defense mechanisms and prevent themselves from destruction. Better understanding how pathogens affect the host immune system will facilitate the development of more effective therapeutic agents against diseases caused by pathogenic microorganisms. This review will be devoted to one of the post-translational modifications, ubiquitination, in connection with host–pathogen interaction. Ubiquitination is a crucial mechanism in numerous cell processes, such as protein degradation, innate immune signaling, protein–protein interactions, and others. Many intracellular pathogens intervene through the ubiquitination system into the host’s innate immune response. Ubiquitination, in addition to acetylation and phosphorylation, is becoming another massively studied modification in connection with host–pathogen interactions. The importance of investigating this post-translational modification in the context of the host–pathogen interaction is constantly increasing, particularly due to the availability of new and sensitive approaches for analyzing ubiquitination.

## 2. Ubiquitin System

Ubiquitination is a significant, reversible post-translational modification managing numerous cellular processes. This modification mediates not only the degradation of proteins but also ensures proper protein function, protein–protein interaction, and subcellular localization [1].

More than 500 proteins possess the ability to recognize ubiquitin (Ub) and Ub-like molecules. These proteins participate in connecting Ub to a specific substrate or its removal from the target molecule. The conjugation and deconjugation of Ub are broadly diverse and complicated processes that regulate many cellular pathways. Deubiquitinating enzymes (DUBs) have recently been attracting greater attention because these proteins are interesting in their function, which is involved in many cellular pathways [2].

**Figure 1:**
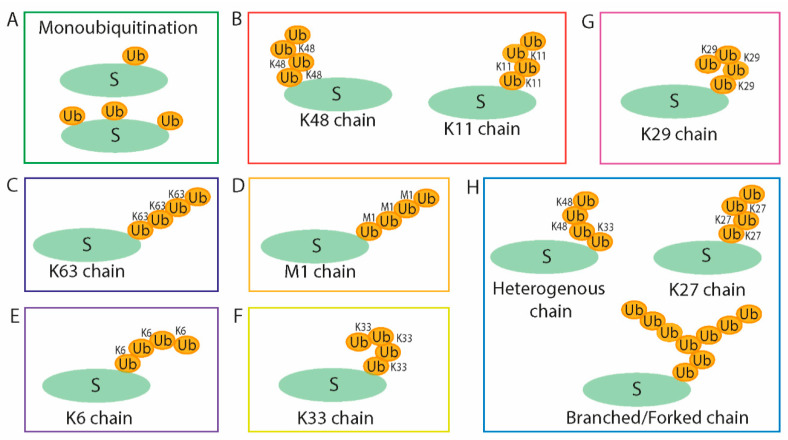
The ubiquitination of substrates may vary in different constructions that play a role in various cell processes: protein interaction and localization (**A**), proteasomal degradation (**B**), NF-κB activation (**C**,**D**), DNA repair (**C**,**E**,**F**), lysosomal targeting (**C**), autophagy (**E**), intracellular trafficking (**F**) and epigenetic regulation (**G**), and many others still unknown functions (**H**). S—substrate, Ub—ubiquitin. Adapted from [5].

Human Ub is a small (8 kDa) and abundant protein consisting of 76 amino acids and comprising as much as 5% of total protein within a cell. This protein is highly conserved among eukaryotic organisms. Monoubiquitination, which is the connection of a single Ub molecule by its C-terminal glycine (G76) to the lysine residue of a substrate protein, influences protein localization, DNA repair, endocytosis, virus budding, and protein–protein interaction [3]. The Ub molecule contains seven lysine residues—K6, K11, K27, K29, K33, K48, and K63—and each of these can be used for the creation of poly-Ub chains by conjugation of Ub molecules in a process known as polyubiquitination. Another type, the M1-linked Ub chain, can be formed by linkage of G76 of the connecting Ub to the N-terminal methionine of a Ub already attached in place of one of the lysine residues. Accordingly, this differential type of Ub connection or creation of poly-Ub chains results in various functions of the modified proteins [4,5]. In general, the ubiquitination can have a degradative or non-degradative purpose. Poly-Ub chains with Ub molecules bound via K48 or K11 determine substrates for proteasomal degradation [6,7]. A Ub chain attached via K63 has a regulatory function in several cell processes as a non-degradative signal, including in DNA reparation, signalization, endocytosis, vesicle transportation, and progression of the cell cycle [3,8]. It has also been reported that K33-linked chains are implicated in intracellular trafficking [9], and, together with K6-linked Ub chains, they are involved in DNA repair [10,11]. Several studies report that K6-linked Ub chains are related to the autophagy process, mitophagy, and xenophagy [12,13]. K29-linked chains have been observed in a proteasome regulation role [14,15] and in other epigenetic regulation in connectivity with the deubiquitinase Trabid [16,17]. The linear M1 ubiquitination is an essential modification in nuclear factor κB (NF-κB) activation [18]. Although the ubiquitin system is being intensively studied, the functions of all types of ubiquitination are not fully understood. Partly, it is because the diverse attachment alternatives give rise to a huge number of a possible complex poly-Ub chain structure (Figure 1).

In addition to Ub itself, other important molecules play vital roles in the ubiquitination processes. These are ubiquitin-like (Ubl) molecules, such as small ubiquitin-related modifier (SUMO), bacterial protein ThiS, and neural precursor cell expressed, developmentally downregulated 8 (NEDD8). Similar to Ub, these are involved in various cellular processes, such as transcriptional regulation, DNA repair, apoptosis, or protein stability [19,20]. Pathogens often target these small molecules during infection because they are involved in critical signaling pathways within the host cell and because disruption of the ubiquitination process could be advantageous to the pathogens [21].

### 2.1. Ubiquitination Process

**Figure 2:**
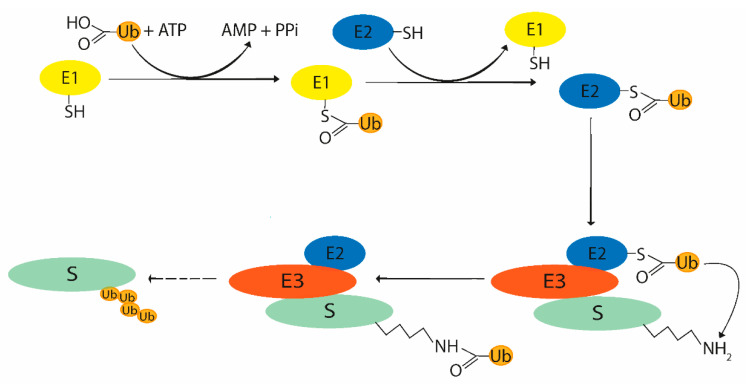
Scheme of ubiquitin binding to the target protein [22]. Ub is activated by ATP-assisted acyl-adenylation, bound to the Ub-activating enzyme (E1), transferred to Ub-conjugating enzyme (E2), and attached to the target protein substrate (S) through Ub-ligase (E3). This cycle mechanism can be repeated for the creation of a polyubiquitin chain. ATP—adenosine triphosphate, AMP—adenosine monophosphate, PPi—pyrophosphate, Ub—ubiquitin. Adapted from [22].

The ubiquitination process consists of a cascade of enzymatic reactions depending on three key enzymes: Ub-activating enzyme (E1), Ub-conjugating enzyme (E2), and Ub-ligase (E3). As mentioned above, ubiquitination is a reversible process, thus similar to some other post-translational modifications, so DUBs are also essential counterparts to these enzymes in the whole process. The initial step involves ATP-dependent Ub activation consisting in acyl-adenylation on the Ub C-terminus. The following part of the process includes transferring Ub to the E2 cysteine active site. The third enzyme, Ub-ligase, is able to recognize the target protein and most typically forms an isopeptide bond between G76 of Ub and lysine of the target protein (Figure 2). The individual key enzymes involved in the ubiquitination system will be described briefly for better understanding of the ubiquitination process.

### 2.2. Enzymes Participating in Ubiquitin System

Many different enzymes are included in the ubiquitin system. Surprisingly, the enzymes and components of the ubiquitination system comprise about 5% of the human proteome [5]. These include two Ub-activating enzymes [23], more than 50 Ub-conjugating enzymes, and several hundred Ub-ligases. The enormous number of different ubiquitination enzymes and varying complexity of Ub chains enable the formation of a subtle and complex regulation system tunable on many regulation levels and consisting of different products [24].

### 2.3. Ubiquitin Ligases

Ub-ligases comprise the biggest group of enzymes involved in the ubiquitination process. Their variability enables their modifying a wide variety of substrates. The primary function of Ub-ligases is transferring Ub to the target protein substrate [4,25]. The Ub ligation enzymes are divided into three families according to the domain each contains: Really Interesting New Gene (RING), homologous with E6-associated protein C-terminus (HECT), and RING-Between-RING (RBR) [26]. RING-type ligases work as scaffolds between the E2 enzyme and the target protein substrate and thus allow ubiquitination of the protein [27]. HECT-type ligases are different from RING-type ligases in the forming of a covalent thioester bond with Ub before its transfer to the substrate [28,29]. RBR-type ligases have both the RING and HECT domains, and their hybrid mechanism of action has been described [30,31,32].

### 2.4. Deubiquitinating Enzymes

The other enzymes playing a vital role in the ubiquitination system are DUBs, which are able to remove Ub from target substrates. DUBs are involved in many cellular processes and pathways, such as gene expression, apoptosis, cell cycle, DNA repair, and cytokine signaling [33]. DUBs were discovered in the group of metalloproteases, and mainly in the group of cysteine proteases. More than 90 DUBs have been identified and described in the human genome [2]. Cysteine DUBs are categorized into six subclasses according to the character of their ubiquitin-protease domains: Ub-specific proteases (USPs); Ub C-terminal hydrolases (UCHs); Machado–Joseph disease protein domain proteases (MJDs); ovarian tumor proteases (OTUs) [2]; a group of enzymes containing motif for interaction with the Ub-containing novel DUB family, known as MINDY [34]; and the newest family, ZUFSP (zinc finger with UFM1 specific peptidase) [35]. DUBs that were found in the metalloprotease group contain a domain that is referred to as JAMM (JAB1/MPN/Mov34 metalloenzyme) [2].

DUBs have widespread mechanisms of action for releasing Ub from the substrate. The Ub molecule is encoded by four genes (*UBC*, *UBB*, *UBA52*, and *UBA80*), and it is expressed as a linear chain of multiple Ub molecules. Subsequently, it has to be cut by DUBs. DUBs are essential for cleavage of polyubiquitin chains and complete removal of Ub chains from ubiquitinated proteins. These mechanisms lead to the maintenance of ubiquitin homeostasis in the cell, reverse Ub signaling, and correction of Ub-protein conjugates [36].

In contrast to other proteases, DUBs are generated as active enzymes. The catalytic activity of DUB cysteine proteases depends on two or three crucial amino acid residues forming a dyad or triad. These essential amino acids are from the group consisting of cysteine, histidine, aspartate, and asparagine. A variety of structures with the catalytic activity of DUBs has been reported [36]. Regulation of DUB activity is mediated by post-translational modifications, allosteric interactions, and subcellular localization. The catalytic triad of some DUBs has to be converted to active conformation by binding to a substrate, otherwise it remains inactive [2,36].

DUBs are able to recognize many ubiquitin-like molecules, isopeptides, and linear peptides, as well as various types of Ub linkage and chain structures. All DUB catalytic domains have a primary Ub-binding domain that encompasses interaction with the distal Ub in a poly-Ub chain. Moreover, each lysine residue in Ub molecule has a unique sequence that can be recognized by DUBs [36].

## 3. Immune Signaling Regulated by Ubiquitination

**Figure 3:**
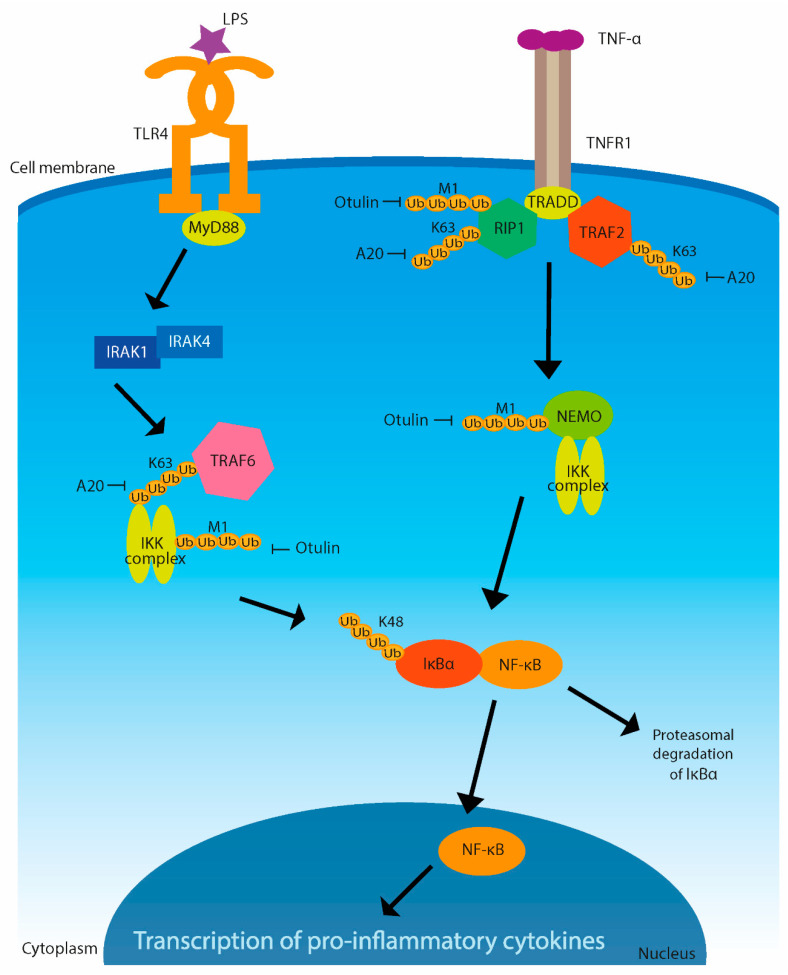
Activation of innate immune signaling pathway by lipopolysaccharide (LPS) and tumor necrosis factor (TNF). LPS is bound by toll-like receptor 4 (TLR4), and then adaptor protein MyD88 interacts with IRAK protein kinases. Recruitment of IRAK complex results in association with another adaptor protein, TRAF6, and this leads to IκB kinase (IKK) activation. NF-κB is a functional transcription factor after IκBα phosphorylation and subsequent dissociation of the complex of NF-κB and IκBα. IκBα, an inhibitor of NF-κB, can also be degraded by way of ubiquitination. Ubiquitinated IκB is degraded in proteasome and NF-κB moves to the nucleus, where it activates the proinflammatory cytokine transcription. A different way of activating the NF-κB pathway shown here is through binding of TNF by TNF receptor 1 (TNFR1). Adaptor protein TRADD recruits receptor-binding protein 1 (RIP1) and TNFR-associated factor TRAF2. RIP1 activates IKK complex and thus also NF-κB pathway. It is shown that A20 and otulin interact with Ub chains in the signaling pathway.

Ubiquitination is one of the crucial mechanisms of inflammation regulation (Figure 3) [37]. The pattern-recognition receptors (PRRs) mediate the recognition of pathogens in the innate immune response. Among them are nucleotide oligomerization domain (NOD)-like receptors, toll-like receptors (TLRs), and retinoic acid-inducible gene-I (RIG-I)-like receptors (RLRs) [38]. All of these receptors are able to induce NF-κB and mitogen-activated protein kinase (MAPK) signaling pathways, which activate the expression of proinflammatory cytokines [39].

One of the major proinflammatory cytokines is tumor necrosis factor (TNF), which induces a strong response and stimulates the recruitment of immune cells to sites of infection or damaged tissue. TNF binds tumor necrosis factor receptor 1 (TNFR1) to trigger a cascade of signaling reactions leading to activation of the NF-κB and MAPK pathways, where Ub linkage is important for NF-κB functioning [40]. K11-, M1-, and K63-linked polyubiquitinations are promoted mainly on receptor-interacting protein 1 (RIP1) but also on TNFR-associated factor TRAF2. This process generates a binding platform for assembly of the distal signaling components, such as cellular inhibitors of apoptosis (c-IAP) [41,42,43]. TNF-stimulated signaling is negatively regulated by DUBs. A DUB known as A20 removes K63-linked poly-Ub chains from RIP1, whereas another DUB, Ub-thioesterase otulin, removes linear chains from RIP1 and NF-κB essential modifier (NEMO), the inhibitor of κB kinase (IKK) regulatory subunit [44,45]. These DUB activities restricting TNF-induced NF-κB and MAPK signaling result in gene expression of proinflammatory cytokines [39].

K63-linked polyubiquitination of signal proteins, such as IRAK-1 or TRAF6, is necessary for triggering immune response based on activation of NF-κB. Polyubiquitination of IRAK-1 and TRAF6 is induced by stimulation of interleukin-1 receptor (IL-1R) or of TLRs by pathogens [46]. If some pathogens are able to block or disrupt this ubiquitination, it gives them the advantage of escaping from the host’s immune response.

NOD-like receptors work as cytoplasmatic PRRs which are able to recognize cytoplasmatic bacterial products. These receptors detect bacterial peptidoglycans. After activation, NODs oligomerize and recruit the kinase RIP2 and CARD9 leading to activation of NF-κB and MAPK signalization by ubiquitination of NEMO [47]. Similar to previous signalizations, the deubiquitinases are involved in the regulation of NOD2-RIP2 activation, such as A20 and otulin [44,48]. NOD2 may play a role in activation of inflammasomes [49]. Mutation in NOD2 can cause autoimmune and inflammatory diseases [47].

Signaling associated with ubiquitination is also observed during overgrowth of microbes that are a common part of the body, and their excessive amount or their metabolite products can influence immune function or dysfunction. Functioning of enzymes associated with ubiquitination is important for self-tolerance in autoimmunity [40]. Ubiquitination is a critical step for the activation of much inflammatory signaling. For example, ubiquitin-induced excessive activation of NF-κB could lead to inflammatory bowel diseases, such as ulcerative colitis or Crohn’s disease, where pathogenic or commensal bacteria in the gastrointestinal tract can be the cause of immune dysregulation [50]. NF-κB is important for intestinal homeostasis and it is regulated by different ubiquitin ligases and deubiquitinating enzymes. In this sense, the ubiquitin-proteasome system is also important, as it is involved in ensuring the homeostasis of post-translational modifications [51]. Moreover, commensal bacterium *Bacteroides fragilis* is able to produce its own eukaryotic-like ubiquitin protein, which is used as bacterial toxin for killing other intestinal bacteria [21,52].

## 4. Host Ubiquitination Machinery as a Target for Pathogens

Intracellular pathogens have developed many molecular mechanisms that are helpful in their strategies, such as their abilities to proliferate intracellularly and to survive host immune responses. Pathogens are able to produce effector proteins affecting the host cell processes in favor of pathogen survival. These proteins, often referred to as virulence factors, either interfere with important host cell structures, such as actin, microtubule, and intermediate filament cytoskeletons, or they are involved in manipulating endocytic, secretory, or signaling pathways in host metabolism [53,54].

Possible mechanisms have been described of bacterial intervention into the host ubiquitin system through the enzyme cascade involving E1, E2, and especially E3 enzymes [55]. Nonetheless, the pathogen interaction in the Ub deconjugation process is not fully understood. In any event, DUBs are enzymes specialized in the cleavage of Ub molecules, and in most cases they do not cleave Ub-like molecules (SUMO or NEDD8). On the other hand, CE clan proteases serve as specific hydrolases of Ub or Ub-like modifications in eukaryotic cells [56]. CE clan proteases are also synthesized by bacteria and work as effector proteins with deubiquitinase, deSUMOylase, or acetyltransferase activity. These effectors have mainly K63-linkage-specific deubiquitinase activity, and they have been found in human pathogens, such as *Salmonella*, *Escherichia*, and *Legionella *[54]. Several known effector proteins produced by bacterial pathogens are described in Table 1.

### 4.1. Bacterial Influence on Host Ubiquitinating and Deubiquitinating Processes

The ability of *Yersinia enterocolitica* and *Y. pseudotuberculosis* to invade host cells depends on the expression and catalytic activity of the human DUB otubain 1 (OTUB1). The main function of OTUB1 is to stabilize the active form of a small GTPase RhoA that is involved in formation of stress fibers during the bacterial attack. *Yersinia* spp. produces multifunctional protein kinase A (YpkA), which interferes with several parts of host cell processes and is regarded as a virulence factor [82]. YpkA is able to interact with post-translationally modified OTUB1 and modulates its influence on RhoA. This effect leads to cytoskeletal rearrangements that could be involved in bacterial uptake [57].

The relationship of the Ub system to bacterial invasion is supported by a recent study that proved the importance of human Ub C-terminal hydrolase UCH-L1 in the entry of *Salmonella enterica *and *Listeria monocytogenes* to the host cell. UCH-L1 is involved in spontaneous formation of actin stress fibers, which facilitate the capture of bacteria and subsequent entry into the cells [83].

The DUB UCH-L5 level is increased in macrophages during *S. enterica *serovar Typhimurium infection, and this leads to greater inflammasome activity. *S. *Typhimurium upregulates the expression of UCH-L5 by an unknown mechanism and causes increased catalytic activity of caspase-1, which limits the IL-1β level. The details of the overall mechanism need to be further investigated. Insufficient production of IL-1β can lead to cell death and the subsequent release of bacteria from macrophages during infection [84].

Pathogenesis of *Helicobacter pylori *is associated with gastritis, peptic ulcer, and gastric cancer. *H. pylori* can affect the host ubiquitin–proteasome system by intervening into the expression and activity of host DUB USP7. A lowering of USP7 decreases the amounts of TRAF6 and tumor suppressor p53, which partly explains the origin of carcinogenesis during chronic infection of *H. pylori* [85].

**Figure 4:**
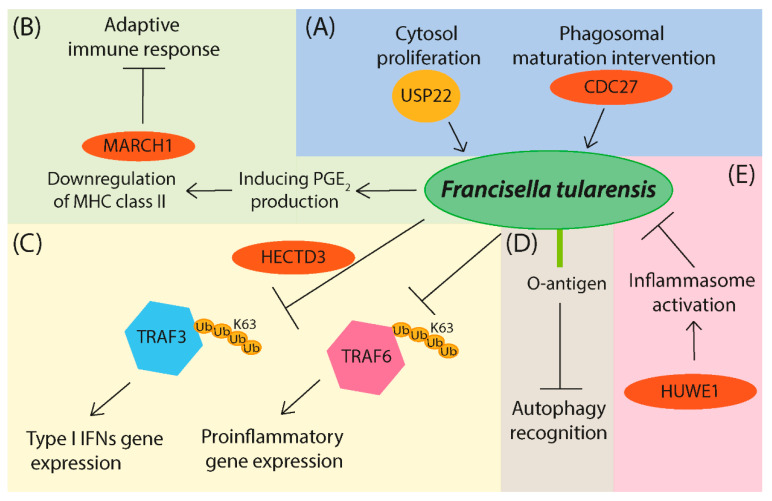
*Francisella tularensis* manipulation of the host cell ubiquitination system. (**A**) *F. tularensis* requires host factor DUB USP22 for cytosol proliferation and E3 ligase CDC27 for intervention to phagosomal maturation. (**B**) Adaptive immune response is changed upon induction of host prostaglandine E_2_ (PGE_2_), where the E3 ligase MARCH1 regulates the ubiquitination of MHC class II. (**C**) Innate immune signalization is subverted by changes in the ubiquitination of TRAF3 and TRAF6 adaptors with E3 ligase HECTD3 regulating the K63 polyubiquitination of TRAF3. (**D**) *F. tularensis* surface O-antigen hinders the autophagy recognition that enables the bacteria proliferation in the cytosol of the host cell. (**E**) Host cell defense mechanism is positively influenced by inflammasome activation through E3 ligase HUWE1.

*Francisella tularensis* is an intracellular pathogen of humans and small mammals causing the zoonotic disease tularemia [86,87]. *F. tularensis* is able to subvert innate immune signaling pathways by influencing K63 polyubiquitination of TRAF3 and TRAF6 (Figure 4) [88]. This process is dependent on a functional type VI secretion system (T6SS). *Francisella* T6SS is encoded by genes located at *Francisella* pathogenicity island (FPI), and T6SS is very important for bacterial virulence. Many critical effector proteins are secreted by T6SS for support of intracellular growth [89], and the whole area of proteins secreted by *F. tularensis* that affect host ubiquitination machinery needs to be explored further. Li et al. [90] observed the function of host E3 ligase HECTD3 during bacterial infection of *Francisella novicida*, *Mycobacterium bovis*, and *Listeria monocytogenes*. The HECTD3 regulates TRAF3 K63 polyubiquitination in innate immune signaling response. Increased host defense against infection upon silencing of HECTD3 ligase has been determined [90]. Another host protein with DUB activity, USP22, is required for the proliferation of *F. tularensis* in the cytosol, and the ubiquitin ligase CDC27 is important for intervention into phagosomal maturation [91]. Guo et al. studied the host defense mechanism of inflammasome activation in *F. novicida* infection. They identified ubiquitin ligase HUWE1 as an AIM2-interacting protein. HUWE1 mediates K27 polyubiquitination of AIM2, and other inflammasomes proteins as NLRP4 and NLRC4, which promotes ASC (Apoptosis-associated Speck-like Protein) speck formation and cleaves caspase-1. Altogether, HUWE1 helps with eliminating bacterial burden, activation of caspase-1, and production of IL-1β [92]. Further, *F. tularensis* disturbs the adaptive immune response by inducing macrophage prostaglandin E_2_ (PGE_2_) production, which directly operates on CD4 T cells by restriction of interferon-γ production [93]. The other effect is in the downregulation of ubiquitin-dependent major histocompatibility complex (MHC) class II expression in macrophages [94]. Francisella-induced PGE_2_ drives the production of soluble macrophage factor FTMΦSN inducing ubiquitination of MHC class II by up-regulation of ubiquitin ligase MARCH1 in macrophages (Figure 4) [95].

Bacterial deubiquitination enzymes or ubiquitin-like proteins may not always be direct effector proteins but may be an essential part of the mechanisms that serve to secrete other effector proteins and virulence factors. As in the case of *Staphylococcus aureus*, where ubiquitin-like protein EsaB has been detected, which is a part of the type VII secretion system and is essential for the secretion activity [96,97].

Oxidative stress is observed during many pathological conditions such as cancer, inflammation, and infection. The ubiquitin proteasome system regulates the nuclear erythroid 2-related factor NRF2 antioxidant response [98] that is part of the KEAP1-NRF2 pathway during the protective response to oxidative stress. The heme oxygenase-1 (HO-1) stabilizes NRF2 during oxidative stress, thus NRF2 is accumulated and the preferential genes for detoxification enzymes are translated [99]. HO-1 has beneficial effects for protection against oxidative injury, apoptosis regulation, or modulation of inflammation. HO-1 as a cytoprotective enzyme with an anti-inflammatory and antioxidant role is very interesting for bacterial and viral pathogens [100,101,102].

### 4.2. Bacterial Modulation of Ubiquitin-Mediated Autophagy during Infection

Autophagy is a lysosome-based degradation process, highly conserved from yeast to human, first termed by Christian de Duve at a conference in 1963 and later in his publication regarding lysosome function [103,104]. Autophagy is activated upon various cellular stresses, such as starvation, organelle damage, or the presence of intracellular bacteria. The main functions of autophagy consist in removing harmful substances (such as protein aggregates, damaged organelles, and intracellular pathogens). Dysfunction of the autophagy process has been associated with numerous diseases in humans, including infectious diseases, cancer, neurodegeneration, cardiovascular disorders, and aging [105,106,107]. The process of autophagy includes the initiation and formation of autophagosomes (double membrane vesicles), subsequent fusion of an autophagosome with the lysosome, and final autolysosome formation that is responsible for the degradation of target molecules [108,109]. Several types of autophagy have been explored, and macroautophagy is the type best described. Macroautophagy is regulated by autophagy-related (ATG) proteins, and other regulators are initiated by formation of the phagophore, where ATG proteins play a critical role. In addition to Ub-independent selective autophagy, where autophagy receptors directly bind to intracellular cargo, Ub-dependent selective autophagy has been described [110]. Target subjects are conjugated with Ub chains and undergo subsequent degradation. Depending upon the target macromolecules, there are different types of autophagy, which include cargos like protein aggregates (aggrephagy), damaged mitochondria (mitophagy), lysosome (lysophagy), and bacteria (xenophagy) [111].

**Figure 5:**
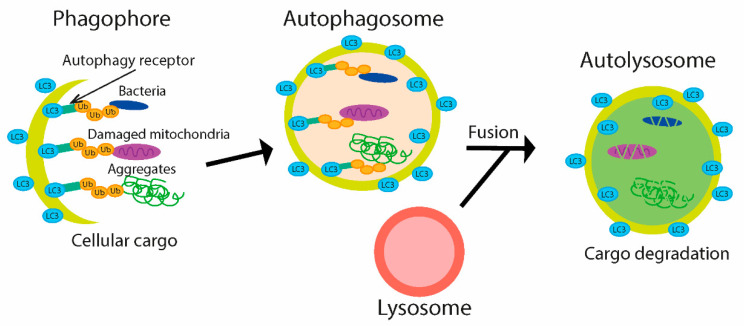
A cargo for selective ubiquitin-dependent autophagy, e.g., bacteria, aggregated protein or damaged mitochondria, is labeled by Ub chain. The cargo is recognized by autophagy receptors on the phagosomal membrane and subsequently maturation leading to the emergence of autophagosome occurs. The autolysosome is formed after fusion of autophagosome with lysosome, where the cargo is degraded. LC3—microtubule-associated protein 1 light chain 3, Ub—ubiquitin.

Not surprisingly, Ub is involved in many processes of autophagy [110,112]. Aggregated proteins, bacteria, and damaged mitochondria are labeled with Ub chains and then are recognized by receptors on the autophagosomal membrane [113]. The initiation of autophagy is dependent on many key regulators, such as ULK1 complex, containing ULK, Atg13, FIP200, and Atg101 proteins. The maturation of autophagosome is dependent on Ub-like conjugation systems, such as microtubule-associated protein 1 light chain 3 (LC3) and the Atg12 systems [111,114]. The ubiquitination of cytosolic bacteria in mammalian cells seems to be necessary for recognition and subsequent degradation by the antimicrobial autophagy process. This interaction is mediated through the LC3-interacting region (LIR) and Ub; subsequently, phagophore and autophagosome are formed, and bacteria are degraded in autolysosome (Figure 5) [110,113].

Many bacteria are able to manipulate ubiquitination during autophagy or even prevent maturation of autophagosome and thus escape from autophagy-dependent degradation. Similar to other intracellular bacteria, *L. monocytogenes* can escape from the phagosome in this way, and it then replicates in the host cell’s cytosol. This step is achieved by quickly expressing virulence factors upon entry to the host cell [115]. *L. monocytogenes* synthetizes ActA protein on its surface, which acts as a shield against autophagy. A mutant strain lacking functioning ActA protein is meanwhile labeled by Ub and unable to escape autophagy-dependent degradation. ActA figures as one of the key virulence factors involved in intracellular motility and is implicated in autophagy evasion. ActA is capable of recruiting actin-related proteins complex, vasodilator-stimulated phosphoproteins, or actin to avoid autophagic recognition by bacterial ubiquitination [77]. Another virulence factor of *L. monocytogenes*, InlK, supports camouflaging the bacterium’s surface by binding host cytoplasmic major vault protein (MVP), thereby blocking ubiquitination of the bacteria such that xenophagy does not occur [78]. Together with another two phospholipases, PlcA and PlcB, *L. monocytogenes* can inhibit autophagosome maturation [116].

*Shigella flexneri* also inhibits the autophagy process. *S. flexneri* produces important proteins that play a role in autophagosomal escape during infection. *S. flexneri* is able to inhibit autophagy by way of VirG and IcsB proteins. During normal conditions, IcsB blocks the binding between bacterial VirG and the host Atg5 (part of a complex that acts as an E3 ligase, through which ubiquitin-like proteins are conjugated to the autophagosomal membrane), thereby leading to reduced efficacy of autophagy. When IcsB is inactivated in a mutant strain lacking IcsB, however, the interaction between VirG and Atg5 occurs, leading to autophagy’s activation [117]. A second role of IscB in inhibiting host immune response against *Shigella* infection by inhibiting a manner of LC3-associated phagocytosis was described later [72]. Additionally, another effector protein, VirA, inactivates Rab1 that is required for the early formation of phagosome [118].

The p62 protein was the first selected autophagy adaptor to be described in mammalian cells [119,120]. It is involved in proteasomal degradation of ubiquitinated proteins [121] and also is involved in autophagy of *S.* Typhimurium. Many substrates are generated by *Salmonella* during infection that are targets for ubiquitination and are recognized by p62, which leads to autophagy of bacteria. *S.* Typhimurium produces SseL, a deubiquitinating enzyme that ensures removal of Ub from bacterial protein products and prevents recruitment of the autophagy markers p62 and LC3 [62]. Other *Salmonella* effectors SseF and SseG are able to impair autophagy initiation via disrupting the Rab1 signaling pathway [122].

*Legionella pneumophila* eliminates host autophagy processes by the production of effector protein RavZ, which hydrolyzes a bond between Ub-like protein Atg8 and phosphatidylethanolamine (PE) [123]. Atg8 (LC3 in mammalian cells) is required for autophagosomal membrane formation, and its conjugation to PE is a critical step in autophagy [124]. Not only does RavZ contain a catalytic domain related to the Ub-like enzymes, it also possesses a phosphatidylinositol-3-phosphate (PI3P) binding domain through which it has the ability to interact with PI3P that is present on the autophagosome surface and is important for RavZ localization to the autophagosomes [125]. A later study proved that RavZ first extracts the LC3-PE complex from the membrane and then acts as a deconjugation enzyme [126]. RavZ deconjugation activity includes not only PE release from LC3-PE but also Ub hydrolysis from substrate [70].

An effector protein important in the bacteria’s evading Ub-mediated autophagy also has been described in the case of *Streptococcus pyogenes*. In contrast to other serotypes, the globally disseminated clone M1T1 of *S. pyogenes* group A is able to replicate in host cells. The M1T1 strain produces SpeB protein, a cysteine protease that is able to degrade the ubiquitin-LC3 adaptor proteins NDP52, p62, and NDR1 [71].

Induction of selective autophagy also occurs in the case of the human pathogen *Staphylococcus aureus*. *S. aureus* was considered to be an extracellular bacterium, but its ability to invade a host cell and replicate inside was observed [127]. *S. aureus* is subject to selective autophagy through labeling by ubiquitination and subsequently it is recognized by receptor proteins SQSTM1, OPTN and CALCOCO2 [128].

*Francisella tularensis* is recognized by autophagic machinery mediated by the ubiquitin-SQSTM1-LC3 pathway after phagosomal escape [129]. *F. tularensis* uses surface polysaccharide O-antigen as masking before autophagy recognition, whereupon bacteria can successfully survive and proliferate in the host cytosol (Figure 4) [130].

The Ub molecule is also essential for the virulence of *Mycobacterium tuberculosis*. As mentioned above, the host immune defense mechanisms use Ub for labeling pathogens to ensure Ub-mediated selective autophagy. Two of the surface proteins of *M. tuberculosis* that directly undergo ubiquitination are Rv1468c [131] and Rv0222 [132]. Deliberate ubiquitination of mycobacterial surfaces and autophagy induction may be a strategy to prevent excessive inflammatory response, allowing bacteria to survive long-term intracellularly [131]. *M. tuberculosis* secretes tyrosine phosphatase PtpA that binds host Ub leading to subsequent activation and works as a dephosphorylating enzyme targeting JNK and MAPK p38 resulting in suppressing innate immunity [133].

Many bacteria modulate host immune response through disruption of autophagy and thus successfully survive and multiplicate inside the host cell. De/ubiquitination, during autophagosome formation, is a critical process, and therefore all effector proteins secreted by bacteria and interfering with autophagy machinery can be potentially relevant for host–pathogen interaction. Below, we focus on effectors directly related to Ub processes.

### 4.3. Bacterial Effectors Intervene with Ubiquitination

#### 4.3.1. The Effect of *Yersinia* on the Host Immune Response

*Yersinia pestis* is the causative agent of plague, while *Y. enterocolitica* and *Y. pseudotuberculosis* cause gastrointestinal diseases. Each of these utilizes the type three secretion system (T3SS) for translocation of virulence proteins into the host cells in order to subvert host response. These effector proteins are classified as *Yersinia* outer proteins (Yops) [134].

The first discovered effector protein with DUB activity was *Yersinia* outer protein J (YopJ) in *Y. pseudotuberculosis* [135]. Its dual functions consist of acetyltransferase and deubiquitinase activity. The acetyltransferase activity leads to inhibition of MAPK and NF-κB signaling pathways, which block the activation of proinflammatory response [58]. The deubiquitinating function of YopJ is used in negative regulation of the signaling pathway by removing Ub from TRAF2, TRAF6, and IκBα. YopJ has shown specificity to K63-linked poly-Ub chains that activate IKK, but it also cleaves K48-linked poly-Ub chains to inhibit proteasomal degradation of IκBα [59]. YopJ is an important virulence factor that is able to directly influence the immune response through deubiquitination activity in *Yersinia* spp. infection.

#### 4.3.2. *Salmonella* Produces Various Effectors

The *Salmonella* genome encodes T3SS to establish itself inside the host cell and translocate numerous effectors to the host cell [136]. Upon entry to the host cell, *Salmonella* is enclosed in a vacuolar compartment termed the *Salmonella*-containing vacuole (SCV) [137].

HECT-like E3 ubiquitin ligase SopA is an effector protein with a regulatory role in host inflammatory response. SopA ligase activity seems to play a crucial role in the induction of enteritis, but the biochemical mechanism and its substrate remain unknown [60].

*S. enterica* serovar Typhimurium produces DUB enzymes SseL and AvrA, which influence virulence and survival of bacteria in host cells [138,139]. AvrA, a homolog of YopJ, has shown a similar function by means of acetyltransferase activity [140]. AvrA removes ubiquitin moieties from IκBα and β-catenin, which regulate the NF-κB and β-catenin signaling pathways. Stabilization of IκBα leads to inhibition of the signaling and thus inhibition of inflammatory response. The stabilized β-catenin increases transcriptional activity, activation of cell proliferation, and inhibition of cell apoptosis [63].

Cysteine protease SseL was found to be secreted through T3SS into the host cell and detected on the vacuolar membrane 6 h after infection. SseL has shown preferences for K63-linked Ub chains on both mono- and polyubiquitin substrates, thus suggesting its involvement in signaling pathways rather than protein degradation [138]. Pruneda et al. studied the cleavage specificity of recombinant SseL, finding that this enzyme is active mainly against K63-linked chains and less so against K48- and K11-linked chains [54]. SseL appears to be necessary for the modulation of host inflammatory response during infection. The deubiquitinase activity of this enzyme leads to suppression of IκBα ubiquitination, which prevents NF-κB activation [61]. A study by Geng et al. showed significantly increased virulence activity of SseL effector-expressing strains in inhibiting the NF-κB pathway during *S. enterica* serovar Pullorum infection in a chicken model [141]. Furthermore, SseL works together with SseJ effector protein and alters the localization of lipid transporter oxysterol binding protein 1 (OSBP1) in *S. enterica* serovar Typhimurium. Deletion of both effectors increases the release of bacteria into the cytoplasm, which proves their important influence on vacuolar stability [142].

#### 4.3.3. *Escherichia* Effectors

Enterohemorrhagic *E. coli* produces E3 ubiquitin ligase effector NleL that is transported to the host cell through outer membrane vesicles (OMVs), where it can ubiquitinylate c-Jun N-terminal kinase (JNK) and thus could have a suppressing effect on the proinflammatory signaling pathway [64].

Intestinal pathogenic *E. coli* produces deubiquitinating enzyme ElaD, which is an ortholog of *S. enterica* serovar Typhimurium SseL effector. ElaD enzyme can interact as deubiquitinase with linear and unanchored K63-linked polyUb chains rather than K48- and K11-linked polyUb chains [54,76]. Thus, the enzymatic activity and biological role of ElaD might be similar to those of *Salmonella* SseL [143].

#### 4.3.4. Miscellaneous Protein Effectors of *Legionella*

The intracellular pathogen *L. pneumophila* also produces effector proteins with deubiquitinating activities that are secreted into the host cell. This pathogen uses a type IV secretion system to translocate effectors into host cells [144].

*L. pneumophila* uses not only canonical but also noncanonical ubiquitination for its infectivity promotion. For this effect, *L. pneumophila* uses SidE family effectors, some of which work as Ub-ligase in a special type of ubiquitination [65,145]. The noncanonical type of ubiquitination connects Ub to serine residues of host substrates via a phosphoribosyl (PR) moiety. It has been shown that PR-ubiquitinated proteins are associated with endoplasmic reticulum remodeling [145]. Moreover, there were two *Legionella*’s effectors (DupA and DupB) acting as deubiquitinase for PR-linked Ub (DUPs) identified. These enzymes counteract the effector SidE with ligase activity and remove PR-Ub from substrates. This process was essential for recruiting fragmented endoplasmic reticulum to *Legionella*-containing vacuoles (LCVs) upon *L. pneumophila *infection [66].

A recent study revealed the structure and mechanism of SdeA-mediated Ub modification [146]. SdeA, a member of the SidE effector family, plays an essential role in bacterial intracellular replication.Its DUB activity was also observed. SdeA has more than one domain and is able to recognize both Ub and NEDD8 Ubl. The N-terminal domain is a key component in the deubiquitination of three different poly-Ub chains: K11, K48, and K63. SdeA also removes K63-linked chains from LCVs, whereby *Legionella* directly and negatively affects the process of autophagy [67]. Downstream domains catalyze the mentioned PR-ubiquitination of substrates [146,147].

Another *Legionella* effector protein is LotA deubiquitinase, a member of the OTU family. LotA consists of two distinctive cysteine protease domains. The first cleavage domain is specific toward K6-linked Ub dimers. The second cysteine catalytic domain mainly contributes to removing chains of K48- and K63-linked Ub. The main function of LotA is to remove Ub from LCVs, and thus it protects bacteria from host immune processes. Not surprisingly, this protein is important for *Legionella’s* optimal intracellular growth [68].

RavD is an *L. pneumophila *effector protein with specific hydrolase activity against linear Ub chains. RavD limits the accumulation of linear Ub chains on LCVs in host cells to inhibit the NF-κB pathway during infection [69]. Surprisingly, RavD also has a phosphatidylinositol-binding domain, which is used to suppress the endolysosomal maturation of LCVs [148].

#### 4.3.5. Insidious *Shigella* Effectors

*Shigella flexneri* is a causative agent of bacterial dysentery, mainly occurring in developing countries. It is able to permeate the epithelial cell wall and subsequently to escape the host’s immune response. After being taken up by macrophages, it is able to induce their apoptosis and escape into the cytoplasm [149]. The bacterium produces an effector protein that can purposefully influence the E2-enzyme level in host ubiquitin machinery. *Shigella* OspG protein is secreted by the T3SS, which can delay the NF-κB signaling pathway, and thus it also decelerates the transcription of NF-κB genes [73].

Proteins from the IpaH family are E3 ligases secreted and translocated into the host cells [150]. The IpaH Ub ligases antagonize activity of the linear Ub chain assembly complex (LUBAC), which acts as a Ub-ligase complex. The LUBAC enzymes generate M1 poly-Ub chains that play a role in the antimicrobial and proinflammatory signaling pathways [151]. IpaH ligases inhibit NF-κB signaling [75,152,153]; they participate in the recruitment of autophagy receptors OPTN, NDP52, and p62; and this helps *Shigella* to escape from xenophagy [74,154].

A DUB similar to *E. coli’s* ElaD and *Salmonella’s* SseL known as SchiCE, is produced by *S. flexneri*. It has the same enzymatic properties and selectively cleaves K63 chains involved in many different cell processes (e.g., innate immune signaling) [54,76].

#### 4.3.6. *Chlamydia* DUB Effectors

*Chlamydia trachomatis* is an obligate intracellular bacterial pathogen that causes various human diseases. *Chlamydiae* are able to avoid alarming the host immune system. One mechanism that *C. trachomatis* uses involves the expression of two enzymes, *Chla*Dub1 and dedicated *Chla*Dub2, with deubiquitinating and deneddylating activities [155]. Pruneda et al. observed that *Chla*Dub1 has a dual function, both K63-deubiquitinase and K-acetyltransferase activities, and, together with *Chla*Dub2, it intervenes in fragmentation of the host Golgi apparatus [156]. Like other intracellular pathogens, *C. trachomatis* suppresses NF-κB signaling activation by inhibiting IκBα ubiquitination and degradation, the actor in this case being *Chla*Dub1 [79]. The *Chla*DUB proteins prevent *Chlamydia*-containing vacuoles’ ubiquitination and stabilize the antiapoptotic protein MCL-1 [157]. Another bacterial DUB, *Chla*OTU, is a member of the OTU family, but its enzymatic specificity toward Ub linkage remains unresolved [80,81].

## 5. Ubiquitination and DUBs in Therapy

Due to importance of ubiquitination and DUBs in the host immune response during infection, these processes could be potential targets in therapies and effective treatments. Therapeutic substances could be helpful for the modulation of inflammatory conditions, antimicrobial and antiviral responses, and so in anticancer therapy. The examples of successful therapy in clinical use are specific inhibitors of proteasomal activity Bortezomib [158,159] or Carfilzomib [160]. They are applied in treatment of multiple myeloma cancer. Furthermore, several potential inhibitors of ubiquitin proteasome system in anticancer therapy are in clinical trials, for example the Nedd8-E1 enzyme inhibitor MLN4924 [161,162] or ubiquitin activating enzyme 1 inhibitor TAK-243 [163]. Nevertheless, no DUB targets have been found for clinical therapeutic use and they are needed to be further elucidated. One of them could be a deubiquitinase inhibitor WP1130, which has anti-infective effect against *L. monocytogenes* or noroviruses [164] and is also used as a treatment of malignancies [165]. The ubiquitin-proteasome system is important for virus replication. Many viruses encode their own DUBs; for example, severe acute respiratory syndrome coronavirus (SARS-CoV) encodes papain-like proteases [166]. Proteasome and DUBs inhibitors could be also helpful in the case of SARS-CoV-2 [167], where they could play an important role in antiviral therapy.

## 6. Conclusions

Ubiquitin and enzymes associated with ubiquitination machinery constitute an important component of signaling pathways in the immune host response to pathogen infection. While, for decades, attention has been devoted to acetylation and phosphorylation, other important post-translational modifications, such as ubiquitination, are now coming into the foreground. Transmitting a signal across the cell is a very intricate and complex process, and all events are more or less interconnected with one another. Effector molecules produced by dangerous pathogens are at the forefront of host immune response modulation. Among these, we find proteins with enzymatic activities that interfere with the ubiquitination process. Through this mechanism, pathogens can be provided with better conditions for survival and their spread within the host organism. When discussing the importance of ubiquitination on the host side, we must also emphasize the ability to deubiquitinate host target proteins on the pathogen side. The pathogen strategy for surviving when interfering with the ubiquitination system may substantially differ for every such pathogen. In some cases, however, bacteria effector proteins function similarly as results from the type of effector enzymatic activity. For instance, SseL, ElaD, and ShiCE effectors produced by *S.* Typhimurium, *E. coli*, and *S. flexneri*, respectively, are all primarily targeted toward K63-linked polyUb and suppress the NF-κB pathway in the innate immune response.

The ability of pathogens to control host immune response to their benefit by means of their effector molecules needs to be further explored. A better understanding of these molecular processes and possible identification of new effectors could be a good source of information for developing new therapeutic agents and vaccines against infections.

## Figures and Tables

**Table 1:** Bacterial effector proteins described in this review.

Effector Protein	Bacteria	Role	Function	References
YpkA	*Yersinia pseudotuberculosis*, *Y. enterocolitica*	Protein kinase	Increasing of bacterial uptake	[57]
YopJ	*Yersinia pseudotuberculosis*	DUB, acetyltransferase	Suppression of MAPK and NF-κB pathway	[58,59]
SopA	*Salmonella* Typhimurium	E3 ligase	Unknown	[60]
SseL	*Salmonella* Typhimurium	DUB	Suppression of NF-κB pathway	[61]
			Inhibition of autophagy	[62]
AvrA	*Salmonella* Typhimurium	DUB, acetyltransferase	Suppression of NF-κB pathway and stabilization of β-catenin	[63]
NleL	*Escherichia coli*	E3 ligase	Suppression of the inflammatory response	[64]
ElaD	*Escherichia coli*	DUB	Suppression of NF-κB pathway	[54]
SidE	*Legionella pneumophila*	E3 ligase	Regulation of phosphoribosyl ubiquitination, cytotoxicity	[65,66]
DupA, B	*Legionella pneumophila*	DUB		
SdeA	*Legionella pneumophila*	DUB	Inhibition of autophagy	[67]
LotA	*Legionella pneumophila*	DUB	Suppression of phagosome maturation	[68]
RavD	*Legionella pneumophila*	DUB	Suppression of phagosome maturation and NF-κB pathway	[69]
RavZ	*Legionella pneumophila*	DUB	Inhibition of autophagy	[70]
SpeB	*Streptococcus pyogenes*	Cysteine protease	Inhibition of autophagy	[71]
IcsB	*Shigella flexneri*	Virulence factor	Inhibition of autophagy	[72]
OspG	*Shigella flexneri*	E2 enzyme	Suppression of NF-κB pathway	[73]
IpaH	*Shigella flexneri*	E3 ligase	Suppression of NF-κB pathway and autophagy	[74,75]
SchiCE	*Shigella flexneri*	DUB	Suppression of NF-κB pathway	[54,76]
ActA	*Listeria monocytogenes*	Virulence factor	Increasing of intracellular motility to avoiding autophagy	[77]
InlK	*Listeria monocytogenes*	Virulence factor	Inhibition of autophagy	[78]
ChlaDUB1	*Chlamydia trachomatis*	DUB, acetyltransferase	Suppression of NF-κB pathway	[79]
ChlaDUB2	*Chlamydia trachomatis*	DUB	Suppression of NF-κB pathway	
ChlaOTU	*Chlamydia pneumonia*	DUB	Unknown	[80,81]
